# The Role of Eye Gaze During Natural Social Interactions in Typical and Autistic People

**DOI:** 10.3389/fpsyg.2019.00560

**Published:** 2019-03-15

**Authors:** Roser Cañigueral, Antonia F. de C. Hamilton

**Affiliations:** Institute of Cognitive Neuroscience, Division of Psychology and Language Sciences, University College London, London, United Kingdom

**Keywords:** eye gaze, audience effect, interpersonal dynamics, social interaction, autism

## Abstract

Social interactions involve complex exchanges of a variety of social signals, such as gaze, facial expressions, speech and gestures. Focusing on the dual function of eye gaze, this review explores how the presence of an audience, communicative purpose and temporal dynamics of gaze allow interacting partners to achieve successful communication. First, we focus on how being watched modulates social cognition and behavior. We then show that the study of interpersonal gaze processing, particularly gaze temporal dynamics, can provide valuable understanding of social behavior in real interactions. We propose that the Interpersonal Gaze Processing model, which combines both sensing and signaling functions of eye gaze, provides a framework to make sense of gaze patterns in live interactions. Finally, we discuss how autistic individuals process the belief in being watched and interpersonal dynamics of gaze, and suggest that systematic manipulation of factors modulating gaze signaling can reveal which aspects of social eye gaze are challenging in autism.

## Introduction

In any face-to-face interaction between two people, both agents are continuously exchanging a variety of social signals, such as gaze, gestures or facial expressions. This two-way exchange of social information is possible because they are able to see each other, and consequently both agents can gather and communicate information. Although traditional cognitive research has largely ignored this interactive nature of social encounters, an increasing number of studies are looking at how social behavior changes in a live interaction, as well as how eye gaze of two individuals coordinates to achieve successful communication, that is, to accurately process incoming signals and send back meaningful signals at a suitable pace.

In the present paper, we explore gaze as a communicative signal in a two-person interaction, considering both patterns of gaze to/from the other person and the interpersonal dynamics of gaze in relation to other behaviors. To explore these issues, we first introduce the dual function of eye gaze and describe two cognitive theories that explain changes in behavior when being watched. We then consider gaze exchanges during communicative situations, and propose the Interpersonal Gaze Processing model as a framework to study the dynamics of gaze in face-to-face interactions. Finally, we look into the case of autism to discuss how studies on the audience effect and interpersonal dynamics of gaze can shed light on why autistic people find social communication challenging.

## The Dual Function of Eye Gaze

Eye gaze has a dual function in human social interaction – we can both perceive information from others and use our gaze to signal to others (Argyle and Cook, 1976; Gobel et al., 2015; Risko et al., 2016). Simmel (1921) already stated that “the eye cannot take unless at the same time it gives.” This contrasts with the auditory modality, where we use our ears to hear, but our mouth to speak. This makes our eyes a powerful tool for social interactions, with a “uniquely sociological function” (Simmel, 1921). For instance, when we see a pair of eyes we can gather information about what other people are looking at (Frischen et al., 2007), and how they feel or think (Baron-Cohen et al., 1997). At the same time, we can use our eyes to strategically cue another’s attention (Kuhn et al., 2009). Depending on the duration and direction of our gaze, we are also able to perceive and signal a variety of meanings, such as desire to communicate (Ho et al., 2015), threat and dominance (Ellyson et al., 1981; Emery, 2000), attractiveness (Argyle and Dean, 1965; Georgescu et al., 2013), or seeking for approval (Efran and Broughton, 1966; Efran, 1968).

The dual function of the eyes has often been ignored in cognitive research studying social interactions. In typical lab studies, participants interact with a monitor that displays pictures or videos of other people, while their gaze or other behavior is recorded (see Risko et al., 2012 for a review). In these experimental settings signals are sent only one-way (from the picture to the participant) and the dual function of gaze is completely lost. Although these traditional approaches allow good experimental control, they are not interactive (Schilbach et al., 2013; Gobel et al., 2015; Risko et al., 2016). Recent research has implemented more ecologically valid approaches that can restore the dual function of gaze. The belief that someone can see us, intrinsic to live interactions, is thought to recruit a range of social cognitive processes that are missing when participants interact with videos or pictures (Risko et al., 2012, 2016; Schilbach et al., 2013). Moreover, in face-to-face interactions communication is multimodal (Vigliocco et al., 2014): information is exchanged through eye gaze, but also through gestures, facial expressions or speech, and all these signals need to be integrated over time and across agents (Jack and Schyns, 2015; Hirai and Kanakogi, 2018; Holler et al., 2018).

In the following, we first describe two cognitive theories that explain changes in behavior when being watched. Then, we discuss why interpersonal dynamics are relevant when studying social eye gaze.

## Cognitive Theories of the Audience Effect

We behave differently when we are alone or in the presence of others. For instance, when we are with other people our actions become more prosocial (Izuma et al., 2009; Izuma et al., 2011), our memory improves (Fullwood and Doherty-Sneddon, 2006), and we smile more (Fridlund, 1991). Triplett first introduced this idea 120 years ago, when he showed that cyclists were faster when competing against each other than against a clock (Triplett, 1898). To explain this effect, he suggested that the “bodily presence of another” causes changes in the behavior of participants, which makes them more competitive when racing against others. However, previous research has shown that there is more than one way in which the presence of another person can change our behavior.

On the one hand, social facilitation refers to a change in behavior caused by the presence of a conspecific that may or may not be watching us (Zajonc, 1965). This effect is present in humans but also in a wide range of species (e.g., cockroaches, rats and monkeys), suggesting that it relies on a simple mechanism like arousal. Zajonc further claimed that an increase in arousal in the presence of others would facilitate dominant behaviors (i.e., responses that are elicited most quickly by a stimulus). For instance in an easy task the dominant response is usually the correct one, while in a difficult task the dominant response is usually the incorrect one. Zajonc and Sales (1966) found that, in the presence of a conspecific, participants performed better on a verbal recognition task with familiar items (easy task), and worse on the same task with unfamiliar items (hard task). This effect has been found in a range of tests on both mental (Geen, 1985) and physical skills (Strauss, 2002). Blascovich et al. (1999) replicated these findings and also showed that, in the presence of others, the cardiovascular system is differently triggered depending on the task: in a difficult task the cardiovascular response fits a threat-like pattern, whereas in an easy task the cardiovascular response fits a challenge-like pattern. This suggests that the facilitation of different dominant responses in the presence of others is mediated by different arousal patterns.

On the other hand, the audience effect is a change in behavior specifically caused by the belief that someone else is watching me. It builds on mechanisms which process the perceptual state of the other, known as perceptual mentalising (Teufel et al., 2010b). Perceptual mentalising modulates the processing of social information from the eyes in a variety of ways. For example, seeing a live-feed of a person with transparent glasses (who can see) leads to a larger gaze cuing effect than a matched stimulus of a person with opaque glasses (who cannot see) (Nuku and Bekkering, 2008; Teufel et al., 2010a), and similar results are seen in tests of visual perspective taking (Furlanetto et al., 2016). This demonstrates that even basic social processing is influenced by the knowledge that another person can see something. The audience effect takes this one step further, considering how our social cognition is affected by the knowledge that another person can see us.

Audience effects differ from social facilitation in that social facilitation could occur if another person is present but looking away, whereas audience effects are specific to the case when another person is believed to be watching (even from another location). When people believe they are being watched, they typically change their behavior to maintain a positive public image. This has been described in terms of self-presentation theory (Bond, 1982), which claims that people modulate their performance in front of others to maintain a good public image and increase their self-esteem. Bond (1982) further showed that making errors while being observed translates into decreased self-esteem and poor performance, regardless of task difficulty.

The audience effect and the dual function of gaze are closely linked in that both require someone who can see us. In line with this, recent evidence suggests that being watched modulates gaze patterns directed at the face of the observer, because in this context direct gaze acquires a social meaning that an individual may or may not wish to signal to someone else. These studies show that in a live interaction people look less to the other person than in a pre-recorded interaction (Laidlaw et al., 2011; Gobel et al., 2015). This change in gaze patterns is further modulated by several factors, such as the observer’s social status (high rank or low rank; Gobel et al., 2015) or role in the interaction (speaker or listener; Freeth et al., 2013; Ho et al., 2015). Thus, when being watched eye gaze is adjusted to send appropriate signals to the observer, rather than to only gather information from the environment.

In the following, we strictly focus on changes in social behavior that derive from audience effects, that is, from the belief in being watched. To explain these changes, two main cognitive theories have been proposed: the Watching Eyes model (Conty et al., 2016) and reputation management theory (Emler, 1990; Resnick et al., 2006; Tennie et al., 2010). Both theories give plausible explanations about the relationship between an individual and an observer, but they have different focus. The Watching Eyes model concentrates on how an observer influences cognitive processing within individuals (self-focus), beyond self-esteem effects proposed by self-presentation theory. Reputation management theory explains how individuals manipulate the observer’s beliefs to their advantage (other-focus) in an updated version of the self-presentation theory. Below we describe each of these theories in more detail.

### Watching Eyes Model

A pair of eyes watching us is an ostensive communicative cue (Csibra and Gergely, 2009) that rapidly captures our attention (Senju and Hasegawa, 2005). Early work on gaze processing proposed various mechanisms how direct gaze modulates our attention and behavior. For instance, Baron-Cohen (1995) suggested that there is a specialized Eye Direction Detector module in the brain. This module rapidly identifies whether we are the target of someone else’s attention by processing the direction of other people’s eyes relative to us. The detection of direct gaze will in turn trigger mentalising processes that allow us to interpret the other person’s mental states (Baron-Cohen and Cross, 1992; Baron-Cohen et al., 1997). Later, Senju and Johnson (2009) coined the term “eye contact effect” to describe changes in cognitive processing following perception of direct gaze, and introduced the Fast-track Modulator model of gaze processing. This model suggests that detection of direct gaze is implemented by a fast subcortical route involving the pulvinar and amygdala, and is modulated by higher cortical regions that depend on social context and task demands. The recently proposed Watching Eyes model (Conty et al., 2016) builds up on these models and suggests that audience effects are due to the “self-referential power of direct gaze.”

Similar to the Fast-track Modulator model by Senju and Johnson (2009), the Watching Eyes model proposes two stages in the processing of direct gaze. In the first stage, direct gaze captures the beholder’s attention by a subcortical route. This seems to be an automatic effect of direct gaze (Senju and Hasegawa, 2005), and is thought to be triggered by the detection of low-level visual cues in eye gaze (e.g., luminance distribution in the eye; von Grünau and Anston, 1995; Kobayashi and Kohshima, 2001). Then, the subcortical route engages mentalizing brain areas (medial prefrontal cortex and temporo-parietal junction) that process the perceptual state of the observer, that is, the belief that s/he is or is not watching us. In the second stage, if the observer can see us, then direct gaze will elicit self-referential processing, and the sense of self-involvement in the interaction will increase. This will lead to the Watching Eyes effects, causing a change in behavior in various ways, such as enhancement of self-awareness (Pönkänen et al., 2011; Baltazar et al., 2014; Hazem et al., 2017) or promotion of prosocial actions (Izuma et al., 2011, 2009).

Recently, Hietanen and Hietanen (2017) have directly tested the Watching Eyes model of self-referential processing. To measure self-referential processing they used the foreign-language task, where participants read sentences in a language that they do not understand and need to match underlined words with pronouns in their native language. In this task, more use of first person singular pronouns is thought to be related to more self-referential processing. Participants completed this task but they watched a video-clip of a person with direct or averted gaze before each sentence was presented. Results showed no effect of eye gaze direction on the pronouns used. Then, a second group of participants completed the same task while they watched live faces with direct or averted face. They found that participants in the direct gaze group used more first person singular pronouns than the averted gaze group. In line with this, a recent study on bodily self-awareness (Hazem et al., 2017) has found that participants are more accurate in rating the intensity of a physiological signal when they believe they are in online connection with someone wearing clear sunglasses (the observer can see through) rather than someone wearing opaque sunglasses (the observer cannot see through). Taken together, these findings show evidence in favor of the Watching Eyes model: to trigger self-reference and self-awareness it is not enough to see a pair of eyes directly gazing at us – the belief that these pair of eyes can see us is also required.

Yet, it is important to consider that different tasks measure different forms of self-reference and self-awareness. This means that different tasks are likely to engage different self-related cognitive processes, which might have different sensitivity to the belief in being watched. For instance, the pronoun-selection task used by Hietanen and Hietanen (2017) is rather intuitive and has been shown to be sensitive to manipulations of self-awareness (Davis and Brock, 1975). However, it could be that other tasks which elicit more complex self-referential cognitive processes (e.g., self-referential effect memory task; Craik and Tulving, 1975; Lombardo et al., 2007) are not as sensitive to this top-down modulation. It is equally important to distinguish between different forms of self-awareness, such as bodily self-awareness (accuracy in reporting physiological signals; Cameron, 2001) and metacognitive self-awareness (accuracy in judging performance in a task; Fleming and Dolan, 2012). Thus, it remains to be seen whether direct gaze and the belief in being watched modulate all forms of self-referential processing and self-awareness or not.

### Reputation Management Theory

Reputation is a social construct that emerges from the desire to cultivate good self-impressions in others (Silver and Shaw, 2018). It is based on how we think others see us, and it changes over time depending on our actions (Izuma, 2012; Cage, 2015). People can gain approval from others and increase their own reputation in various ways, such as acting for the benefit of other people or behaving according to social norms. To maintain or manage reputation, individuals need to think about what others think of them, care about how others see them, and have the desire to foster positive impressions in others (Izuma, 2012; Cage, 2015). Thus, mentalizing and social motivation have a central function in reputation management (Saito et al., 2010; Tennie et al., 2010; Izuma, 2012; Cage, 2015). In line with this, neuroimaging studies have shown that mentalizing and reward brain areas are engaged during different phases of reputation management, such as processing what others think of them (e.g., medial prefrontal cortex; Frith and Frith, 2006; Izuma et al., 2010) or anticipating positive reputation (e.g., ventral striatum; Izuma et al., 2009, 2010) respectively.

One strategy that people use to maintain a good reputation in front of others is to behave in a more prosocial fashion (Smith and Bird, 2000; Bradley et al., 2018). A way to measure prosocial behavior in the lab is by using economic games. Because they usually have repeated trials, this facilitates reputation building between participants in the game (Pfeiffer and Nowak, 2006; Bradley et al., 2018). For instance, Filiz-Ozbay and Ozbay (2014) used the Public Goods game and found that people invest more effort to contribute to public, but not private, goods when someone is observing them. Izuma et al. (2011) used the Dictator game (Kahneman et al., 1986; Guala and Mittone, 2010) as a donation task, where participants receive a sum of money and must decide on repeated trials whether to accept a proposal to share the money with a charity, or reject it and keep all the money. Results showed that in the presence of a confederate who pretended to monitor the answers, participants decided to accept the proposed sharing more often than when they were alone in the room. These findings clearly illustrate how participants manipulate the beliefs of the observer to maintain their good reputation.

Several factors modulate how strong the audience effect is on prosocial behavior (Bradley et al., 2018), such as the identity of the observer (experimenter, other participants, stranger) or whether decisions of participants are consequential. For instance, Cage et al. (2013) also used the Dictator game in the presence and absence of a confederate, but additionally contrasted two conditions: one in which participants believed the recipient of the sharing arrangement was an individual who could later reciprocate (consequential decision), and one in which the recipient could not reciprocate (non-consequential decision). They found that participants accepted the chance to share money most frequently in the presence of a confederate and when the confederate could later reciprocate. This shows that the context associated with the observer (e.g., can s/he reciprocate or not?) also modulates the extent to which being watched affects behavior.

Another strategy used to maintain reputation is to behave according to social norms. Social norms can be of various kinds, such as saying thank you or holding a door for someone after you. A more subtle type of social norm is civil inattention (Goffman, 1963), which proposes that the amount of gaze directed to strangers “should be enough to acknowledge their presence but not so much as to indicate that they are of special interest.” Multiple studies have used eye-tracking to test if social attention is modulated according to social norms of eye gaze. For instance, Laidlaw et al. (2011) found that participants sitting in a waiting room would look more to a confederate in a video-clip than to the same confederate present in the room. The authors claimed that this change in gaze patterns is due to a social norm whereby it is not polite to stare at someone, which in turn translates into active disengagement.

Some of these studies also show that gaze patterns in live contexts are modulated by a number of factors that do not have any effect when participants watch video-clips. Gobel et al. (2015) found that participants spend more time gazing at video-clips of a low rank confederate and less time gazing at video-clips of a high rank confederate, but only when they believe the confederate will later see their gaze recording. These two gaze behaviors, direct and averted gaze, have been associated with signalling of dominance and submission, respectively (Ellyson et al., 1981; Emery, 2000). In another study, Foulsham et al., 2011 showed that participants gaze less to close pedestrians than distant pedestrians to avoid appearing as an interaction partner to strangers (see also Argyle and Dean, 1965; Gallup et al., 2012). These studies indicate that, when an observer is watching, eye gaze acquires a signaling function and this will subtly modulate gaze patterns to send appropriate signals to the observer. Moreover, the social skills of participants and their looking behavior are correlated in live but not lab settings (Laidlaw et al., 2011). This suggests that individuals who successfully interact with other people are those who can modulate social behavior according to requirements of the social context.

So far, we have discussed how the presence of an observer modulates an individual’s cognitive processing, both self-focused (Watching Eyes model) and other-focused (reputation management theory). However, the studies presented above have a major limitation: confederate and participant are not expected (and do not intend) to interact, verbally or physically, with each other. This means that there is no explicit communicative exchange between them. In the same way that social behavior changes when participants watch a video-recorded person or a live person, it could be that it also differs between a situation where there is potential for an interaction and a situation where there is an actual interaction with explicit communicative exchanges (henceforth communicative encounter; Foulsham et al., 2011; Wu et al., 2013; Macdonald and Tatler, 2018). Focusing on the particular case of eye gaze, in the next section we argue that interpersonal gaze dynamics have a key role in modulating social behavior during communicative encounters.

## Interpersonal Dynamics of Eye Gaze

Original studies about the role of eye gaze during communicative encounters date back to the 60 s, when Argyle and colleagues (Argyle and Cook, 1976; Argyle and Dean, 1965) put forward the intimacy equilibrium model, which is the first account on the relationship between “looking and liking:” they showed that gaze directed at other people serves to control the level of intimacy or affiliation with the partner, and that it compensates with other behaviors (e.g., physical proximity) to achieve an equilibrium level of intimacy (see also Loeb, 1972). Furthermore, Watzlawick et al. (1967) proposed the idea that “one cannot not communicate,” since the lack of response is a response in itself (e.g., not looking at someone signals lack of interest in the interaction; Goffman, 1963).

Recent studies show that direct gaze can act as an ostensive communicative signal (Csibra and Gergely, 2009). During face-to-face interactions, where individuals exchange information with communicative purpose through a variety of channels (e.g., gaze, gestures, facial expressions, speech), direct gaze helps to integrate and coordinate auditory and visual signals (Bavelas et al., 2002). Moreover, it has been shown that to successfully produce and detect gestures with communicative purpose, information conveyed by gaze signals (e.g., direct gaze) is preferentially used over information conveyed by kinematics of the gesture (Trujillo et al., 2018). Thus, eye gaze has a core function in leading social interactions up to successful communicative exchanges, where there is efficient transmission of information between sender and receiver.

In the studies presented in the previous section, the authors claim that changes in eye gaze when participants are being watched respond to demands of social norms (Foulsham et al., 2011; Laidlaw et al., 2011; Gobel et al., 2015). The context of those studies does not require participants to explicitly communicate with the confederate, but only look (or not) at each other. Moreover, the confederate is usually a complete stranger to the participant. It is therefore not surprising that this awkward interaction without communicative purpose leads participants to modulate eye gaze in compliance with social norms (Wu et al., 2013). However, in communicative encounters (e.g., conversation) gaze patterns need to coordinate with other verbal and non-verbal signals to successfully receive and send signals (Bavelas et al., 2002; Trujillo et al., 2018). In studying such communications, we must consider not just the average pattern of gaze (toward/away from the face) but also the dynamics of gaze behavior in relation to other social events (speech, turn taking, facial expressions, etc.). This means that to succeed during communicative exchanges, eye gaze needs not only modulation by social norms, but also constant adjustments to keep pace with interpersonal dynamics that emerge as the interaction develops.

In the following, we first describe the main social functions that eye gaze has during communicative interactions. Then, we focus on the temporal dynamics of gaze as a key mechanism that enables meaningful interpersonal exchanges during communication, as well as successful progression of the interaction.

### Social Functions of Eye Gaze During Conversation

During communicative encounters, such as conversations, the eyes of both agents are generally very active. In a seminal study on gaze direction during conversation, Kendon identified asymmetrical gaze behavior between speakers and listeners (Kendon, 1967): while listeners gazed at speakers most of the time, speakers shifted their gaze toward and away from listeners. More recently, Rogers et al. (2018) found that during a 4 min conversation participants spent on average 60% of the time directing their gaze toward the face of the other person (only 10% of the time it was directed specifically to the eyes), and that these events were approximately 2.2 s long (for direct eye contact events were 0.36 s long). The brief duration of these events supports Kendon’s original findings, because it indicates that participants are constantly alternating their gaze between face or eyes of their partner and other regions. There has been much debate about the meaning of these rapid and subtle changes in eye gaze direction and duration. Kendon (1967) originally suggested that they give rise to three main social functions of gaze. Note that, although the gaze patterns described below allow us to send signals to another person, these signals are sent implicitly and without awareness.

First, he proposed that eye gaze has a regulatory function during conversation, because it allows individuals to modulate transitions between speaker and listener states (i.e., turn-taking). In line with this, it has been found that speakers use averted gaze when they begin to talk and during hesitation (probably to indicate that they want to retain their role as speakers), but they use direct gaze to the listener when they are about to end an utterance (probably to signal that their turn is ending and that the listener can take the floor) (Kendon, 1967; Duncan and Fiske, 1977;Cummins, 2012; Sandgren et al., 2012; Ho et al., 2015). However, as noted by Ho et al. (2015) conversation is a two-way process and this means that the listener is also responsible to regulate in turn-taking. For instance, it has been shown that listeners make more gestures, head shifts and gaze shifts before speaking, probably to indicate to the speaker that they want to take the turn (Harrigan, 1985).

Second, Kendon suggested that eye gaze has a monitoring function: it allows each participant to track attentional states and facial displays of the partner to ensure mutual understanding and seek social approval from others (Efran and Broughton, 1966; Efran, 1968; Kleinke, 1986). Indeed, speakers try to gain more information about what listeners think by engaging in brief periods of mutual eye gaze, which elicit back-channeling (i.e., listener’s brief responses showing comprehension of what the speaker is saying) (Bavelas et al., 2002). Rogers et al. (2018) have also proposed that brief and rapid gaze shifting between gaze directed to the eyes and to other facial regions (e.g., mouth, eyebrows) may serve to scan facial features and pick subtle cues that help interpreting the meaning of what is being said. The monitoring function of gaze can also have high cognitive costs. For instance, when participants are asked to look at the face of the experimenter, they perform worse than participants who can avert their gaze naturally (Beattie, 1981), or who are asked to fixate on other static or dynamic stimuli (Markson and Paterson, 2009). Thus, Kendon also claimed that speakers avert their gaze partly to reduce the costs associated with monitoring a face.

Third, Kendon proposed that eye gaze has an expressive function, which allows participants to regulate the level of arousal in the interaction. He found that some participants tended to avert their gaze at moments of high emotion, and that the amount of eye contact was inversely related to the frequency of smiling. He suggested that averting gaze at these highly emotional moments could be interpreted as a “cut off” act to express embarrassment and reduce arousal. Moreover, the expressive function of mutual eye gaze has been associated with affiliation and attraction (Argyle and Dean, 1965; Argyle and Cook, 1976; Georgescu et al., 2013), with dominance and power (Ellyson et al., 1981; Emery, 2000; Gobel et al., 2015), and more recently with expressing response preference to polar questions (Kendrick and Holler, 2017).

It is important to bear in mind that the social functions of gaze are only meaningful during face-to-face interactions, where both partners can see each other. It is only in this context that eye gaze has a dual function and both agents can perceive and signal information (Gobel et al., 2015; Risko et al., 2016). Moreover, gaze signals are not isolated: speakers need to shift their gaze toward or away from the listener at specific time points during speech, listeners need to coordinate gaze direction with facial expressions to indicate preference or reduce arousal, and speakers and listeners need to engage in brief mutual gaze periods to exchange turns or elicit back-channeling. Thus, to succeed in communicative encounters social signals need to be coordinated within and across conversation partners over time.

### Temporal Dynamics of Gaze

Successful communication requires that both agents involved in the interaction process incoming signals and send back meaningful signals at a suitable pace. Since these signaling exchanges (specially for eye gaze) happen very quickly, timing becomes a critical factor to enable successful progression of the interaction. The need for timed coordination gives rise to patterns of gaze behavior, that is, temporal dependencies that emerge between gaze and other social signals. For instance, using gaze cueing paradigms (e.g., Posner’s paradigm; Posner, 1980) it has been shown that averted gaze results in reflexive gaze following behavior, which is key to build joint attention (Pfeiffer et al., 2013). Similarly, there could be a systematic relationship between gaze and speech within an individual (e.g., direct gaze at others when finishing an utterance, but avert gaze when hesitating; Ho et al., 2015), or between the gaze direction of two conversation partners (e.g., establish mutual eye gaze to elicit back-channeling; Bavelas et al., 2002). The presence and direction of these temporal dependencies at different time points can contribute to identifying which social cognitive processes modulate gaze behavior in the course of the interaction.

Experimentally manipulating temporal dynamics of eye gaze in the lab can be challenging, because it requires some degree of control over gaze patterns for at least one of the agents. Virtual reality and humanoid robot avatars offer an efficient alternative to this issue, because their behavior can be meticulously controlled while participants respond with comparable social behaviors as in interactions with real human beings (Pfeiffer et al., 2013). With the aim of studying interactions in a truly reciprocal context, Wilms et al. (2010) created the now widely used gaze-contingent eye-tracking paradigm (see also Bayliss et al., 2012; Kim and Mundy, 2012; Edwards et al., 2015). In this paradigm, participants wearing an eye-tracker interact with an avatar whose gaze is controlled by the real-time gaze data collected from the participant. Thus, the avatar becomes a gaze-contingent stimulus that responds to the participant’s gaze behavior. Using this paradigm in the context of joint attention, it has been shown that avatars are perceived as more human-like (Pfeiffer et al., 2011) and more likeable (Grynszpan et al., 2017; Willemse et al., 2018) if they follow the gaze of participants to achieve joint attention. Another study has shown that participants are quicker to assume that the avatar understands their instructions when there is contingent gaze following (Frädrich et al., 2018). At the neural level, joint attention has been linked to activation in brain areas related to gaze direction (superior temporal sulcus), processing of reward (ventral striatum) and mental states (medial prefrontal cortex, temporo-parietal junction) (Pelphrey et al., 2004; Schilbach et al., 2010; Pfeiffer et al., 2013; Caruana et al., 2015).

Some attempts have also been made to study the nature of temporal dynamics of gaze in real human-to-human interactions. For instance, Lachat et al. (2012) designed a joint attention task where dyads of participants engaged in joint and no-joint attention periods, respectively. They found that during joint attention periods mu rhythms in centro-parietal regions were suppressed for both leaders and followers, which has been previously associated with interpersonal coordination processes (Naeem et al., 2012). In another study, participants completed a structured interview with a pre-recorded or live confederate, whose gaze was directed at them or averted (Freeth et al., 2013). They found that participants gazed more to the confederate’s face if her gaze was directed at them than if her gaze was averted, but only in the live condition. This means that participants’ gaze was adjusted according to the looking behavior of the confederate only when their gaze acquired a signaling function (i.e., they were in a live interaction), thus creating a reciprocal social signal. Recently, a dual eye-tracking study (Macdonald and Tatler, 2018) has also shown that pairs of participants who are given specific social roles in a collaborative task align their gaze quicker than pairs who have no social role. This indicates that eye gaze adjusts to the communicative purpose embedded in different social contexts.

Gaze dynamics are fundamental to efficiently communicate with other people, that is, to enable information transfer between individuals. It has recently been suggested that brain-to-brain coherence (i.e., synchronization of neural activity between two brains) provides a marker of the success of a communication between two people (Hasson et al., 2012), and several hyperscanning studies show that mutual gaze triggers neural coherence between partners. For instance, mutual gaze mediates neural coupling between parents and infants, which has been associated with appropriate use of communicative signals according to each social context later in development (Piazza et al., 2018). Neural coherence between parents and infants has been shown to be stronger in live versus pre-recorded interactions (Leong et al., 2017). Moreover, in a joint attention task through a video-feed, moment of eye contact was characterized by increased synchronization of frontal brain activity between participants (Saito et al., 2010). Hirsch et al. (2017) have also shown that only when partners in a dyad make eye contact (compared to when both partners look at a photograph of a face) brain-to-brain coherence between partners’ increases in regions associated with processing of social information (temporo-parietal and frontal regions). These findings suggest that direct gaze acts as a signal that enhances the temporal alignment of two brains (Hasson et al., 2012; Gallotti et al., 2017), thus facilitating the sharing of information.

All these studies show that temporal coordination of gaze patterns are characteristic of human interactions (Pfeiffer et al., 2011; Willemse et al., 2018), and that they have beneficial effects for the interacting partners, such as increasing the reward value of the interaction (Schilbach et al., 2010), or facilitating social coordination (Lachat et al., 2012; Freeth et al., 2013; Frädrich et al., 2018) and information transfer (Saito et al., 2010; Hirsch et al., 2017; Leong et al., 2017). They also highlight that gaze is a dynamic and interpersonal signal which changes over time depending on the social situation and communicative purpose. However, there is no cognitive model of gaze processing that takes into account these interactive factors. We believe that in the current context of social cognitive research, which has a strong focus on ecologically valid approaches (Schilbach et al., 2013; Risko et al., 2016), there is an urgent need to build up a cognitive model of eye gaze in live interactions. With this aim, in the next section we introduce the Interpersonal Gaze Processing model, which tries to makes sense of gaze dynamics during face-to-face interactions.

## Interpersonal Gaze Processing Model: Active Sensing and Social Signaling

The dual function of the eyes means that our gaze both gains information form the environment and signals information to others. Early cognitive research already described how the visual system gains information from the environment in non-social contexts (Koch and Ullman, 1985; Itti and Koch, 2001). However, to our knowledge there is no cognitive model of gaze processing in social contexts. Here we draw on two distinct frameworks, from motor control (active sensing; Yang et al., 2016) and from animal communication (signaling theory; Zahavi, 1975; Grafen, 1990), to introduce the Interpersonal Gaze Processing model. This model considers how these two frameworks can be combined in the domain of social gaze to take into account both its sensing and signaling functions. In the following, we describe how active sensing and signaling theory are useful to explain gaze behavior.

### Active Sensing in Eye Gaze

Active sensing is a key process in our interaction with the world, since it allows our sensors to be directed to the environment in order to extract relevant information (Yang et al., 2016). Gaze behavior (i.e., deciding where to look) can be considered a form of active sensing in that we choose to move our eyes to specific locations to sample useful information from a visual scene. Since our visual system only gains high-resolution information for items falling in the fovea, the motor system needs to move our eyes to orient the fovea to different locations of interest. Thus, our motor actions shape the quality of the sensory information we sample (Yang et al., 2016).

**FIGURE 1:**
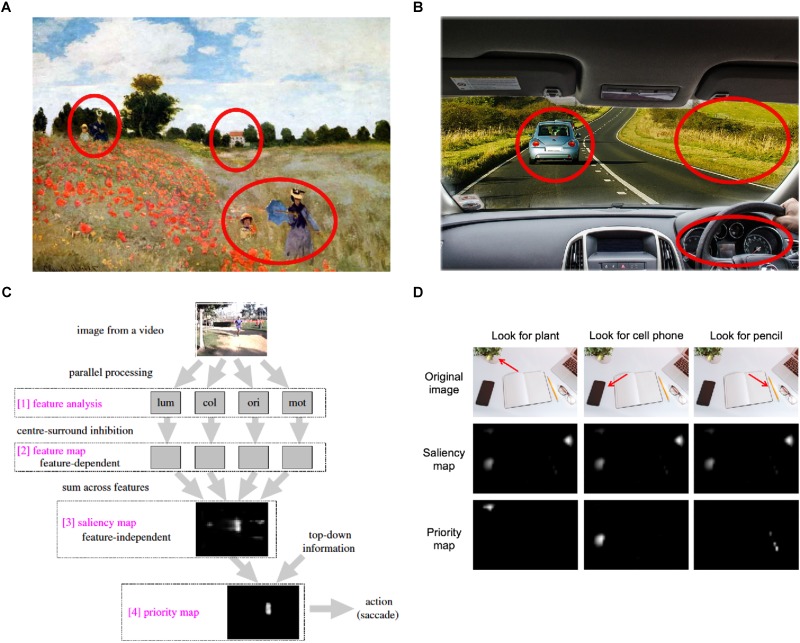
**(A,B)** Sample visual scenes with red circles indicating different locations where gaze can be directed. Photographic reproduction of painting “Poppies” by Claude Monet **(A)**, and original image published by Max Pixel under the Creative Commons CC0 License **(B)**. **(C)** Feature, saliency and priority maps (original image published by Veale et al., 2017 under the Creative Commons Attribution License). **(D)** Priority maps for different task goals [original image published by Max Pixel under the Creative Commons CC0 License; maps were obtained with Saliency Toolbox for Matlab (Walther and Koch, 2006)].

The active sensing framework provides a mathematical account of how we can sample the world with our eyes to get useful information. Because we can only direct our eyes at one location at a time, each eye movement (i.e., saccade) comes at some opportunity cost. For instance, in Figure 1A, looking at the woman and child on the bottom means we might lose the chance to get information about the house in the center or the woman and child on the left. Similarly, in Figure 1B, looking at the landscape on the right means we will lose information about the blue car on the left or the speedometer. Active sensing suggests that saccades are planned to maximize the information we sample depending on the goal of the task at hand.

To understand how sampled information is maximized it is useful to consider the concept of saliency maps. A saliency map is “an explicit two-dimensional topographical map that encodes stimulus conspicuity, or saliency, at every location in the visual scene” (Itti and Koch, 2001). It results from the combination of different topographical or feature maps, each representing a single visual feature (Koch and Ullman, 1985; Itti et al., 1998; Veale et al., 2017), such as intensity or color. A saliency map is a pre-attentive computation, in the sense that at this stage all locations are competing for representation in the visual cortex (Itti and Koch, 2001). Only the location that is most salient will gain further access in downstream visual areas and the oculomotor nerve, and guide the next eye movement so as to deploy attention in that specific location (Koch and Ullman, 1985; Kastner and Ungerleider, 2000; Itti and Koch, 2001; Veale et al., 2017) (see Figure 1C).

Early models of saliency maps only included static features of visual scenes (e.g., color, orientation, intensity, center-surround difference; Koch and Ullman, 1985; Itti and Koch, 2001), but later proposals have suggested saliency maps that also integrate dynamic features (Milanese et al., 1995; Jeong et al., 2008). For instance, the integrated saliency map by Jeong et al. (2008) considers dynamic features such as rotation, expansion, contraction or planar motion. These dynamic features are especially effective in attracting visual attention, and have been associated with an alerting mechanism that rapidly detects moving objects (Milanese et al., 1995). Both static and dynamic features generate a bottom-up bias on the saliency map.

However, saliency maps can also be modeled by a top-down bias emerging from affective features (e.g., preference or dislike for the visual stimuli; Olshausen et al., 1993; Tsotsos et al., 1995; Itti et al., 1998; Veale et al., 2017; Jeong et al., 2008) (see Figure 1C). Affective features are mainly associated with the goal of the task at hand, and are integrated with bottom-up information in associative visual areas (extrastriate cortex) (Veale et al., 2017). For instance, as shown on Figure 1D, different search goals will model different priority maps derived from the same saliency map. Recent evidence has also found that when participants view social naturalistic scenes low-level salient features are less important, and participants primarily fixate on the faces and eyes of people in the scene (Nasiopoulos et al., 2015; End and Gamer, 2017; Rubo and Gamer, 2018). This suggests that there is an implicit preferential bias to attend to others in social scenes to obtain information about them (Nasiopoulos et al., 2015). In the same way that non-social task goals (e.g., search for the cell phone) model different priority maps, implicit social task goals (e.g., identify feelings of an actress in a movie) will model different sensing maps. This top-down bias is particularly important in the context of active sensing, since the task goal will modify the reward value of each location in the visual scene and, in turn, determine which information needs to be maximized (Jeong et al., 2008; Yang et al., 2016).

Active sensing provides a useful framework to understand how eye movements are planned to process non-social stimuli (e.g., objects or landscapes), as well as social stimuli in pictures or videos. In both cases, the saccade planner combines bottom-up and top-down features in a priority or sensing map to maximize information relevant for the task and decide where gaze is next directed (Yang et al., 2016). However, in the case of face-to-face interactions, our gaze not only needs to maximize the information gained but also send signals to another person (i.e., dual function of eyes; Argyle and Cook, 1976; Gobel et al., 2015; Risko et al., 2016).

### Social Signaling and Eye Gaze

Research on animal communication has explored in detail the question of what behavior counts as a social signal and what message (if any) is sent (Stegmann, 2013). A cue is a behavior or feature that can be used by another creature to guide its behavior; for example, mosquitoes use the increased carbon dioxide in exhaled air as a cue to find people to bite, but there is no benefit here to those sending the cue. In contrast, the mating call of a bird that attracts a mate acts as a signal because it benefits both sender and receiver (Stegmann, 2013). A key way to distinguish between these is that signals are sent with the purpose of having an effect on another individual, which means they are more likely to be sent when they can be received. In the context of human interaction, signals are sent when another person is present (an audience effect) but should not be sent when a person acts alone. A stronger definition of explicit and deliberate signaling might require sending a signal repeatedly or elaborating on the signal until it is received. However, based on animal communication models (Stegmann, 2013), we will use a minimal definition of communication where signals are sent implicitly.

As described above, our eyes can act both as a cue to our current thoughts (e.g., if I am looking at my watch, I want to know the time) and as a signal to another person (e.g., I ostentatiously stare at my watch to signal to my friend that we must leave the party) (Argyle and Cook, 1976; Gobel et al., 2015; Risko et al., 2016). As Watzlavick’s axiom “one cannot not communicate” (Watzlawick et al., 1967) suggests, even in a waiting room where two people are not intended to communicate and avoid engaging in eye contact, they are sending a signal that means “I do not want to interact with you” (Foulsham et al., 2011). This means that, in line with signaling theory, in face-to-face interactions our eye movements are constantly planned so as to send signals to others, and not just to gain information from the world. We propose that the signaling function of gaze creates a signaling map in the brain equivalent to the sensing map generated by the sensing function. In the same way that sensing maps show where to look to gain information, we hypothesize that signaling maps are computed in the brain to show where to look to send an appropriate signal to another person. In the following, we argue that the signaling map is computed by taking into account three key factors: communicative purpose, other’s gaze direction, and coordination with other social signals.

First, the value of each gaze target in the signaling map will vary depending on the communicative purpose, that is, the type of message we wish to send. Just as saliency maps incorporate the task goal to create priority or sensing maps of visual attention, signaling maps need to take into account the communicative purpose. Imagine a waiting room with two people, where one person (A) wants to engage in an interaction, but the other person (B) does not. For person A, the optimal signaling behavior is to direct gaze to person B in order to send the message “I want to engage in an interaction with you.” However, person B should avert gaze to efficiently signal “I do not want to interact with you.” Thus, the signaling map will be different for person A and B, depending on the message they want to send.

Second, the signaling map will change according to the direction of the other person’s gaze. The relationship between other’s gaze direction and the signaling map lies in the fact that my signal will be received depending on whether the other person is gazing at us or not. Let’s go back to the case of the waiting room with person A and B. For person A, who wishes to interact with person B, the optimal signaling behavior is to direct her gaze when person B is also looking at her, in order to disclose interest in the interaction. Directing her gaze when B is not looking has little benefit, because the signal will not be received. Equally, for person B the optimal signaling behavior is to avert gaze specifically when A is looking at her. This illustrates how the values associated with each location in the signaling map changes on a moment-by-moment basis, contingent on the gaze direction of the other person and in relation to communicative purpose.

Finally, the signaling map depends on the need to coordinate with other social signals that are sent in multimodal communication, such as speech or gestures (Vigliocco et al., 2014; Ho et al., 2015; Jack and Schyns, 2015; Hirai and Kanakogi, 2018; Holler et al., 2018; Trujillo et al., 2018). This is particularly relevant for explicit communicative encounters. Imagine that person A and B in the waiting room are now engaged in a lively conversation: to signal interest in keeping the conversation going, the choice of direct or averted gaze will vary depending on the role of each partner in the conversation, as well as the time-course of speech itself. For instance, when person A starts speaking, she may avert gaze every now and then to signal she still has more things to say (Kendon, 1967; Ho et al., 2015). While person B is listening, her gaze may be directed toward person A in order to signal interest in what A is saying (Kendon, 1967; Ho et al., 2015). However, when person A is finishing the utterance, she may look toward person B to signal that she can take the floor (Kendon, 1967; Ho et al., 2015). Thus, the coordination with other social signals also modulates the optimal location in the signaling map on a moment-by-moment basis.

Signaling theory provides a framework to understand how the communicative function of gaze shapes the planning of eye movements during face-to-face interactions. In the following, we propose a model where both active sensing and social signaling are combined to make sense of gaze patterns in human-to-human communication.

### The Interpersonal Gaze Processing Model

**FIGURE 2:**
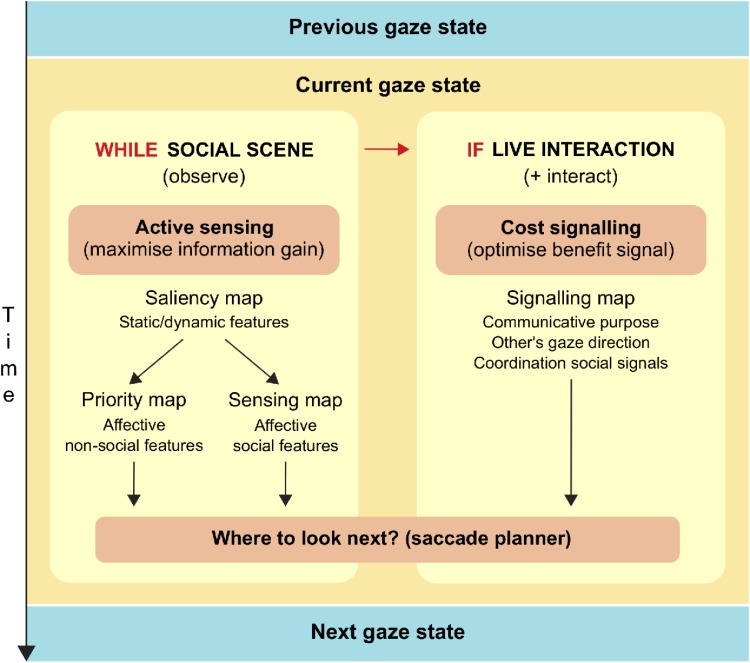
Diagram summarizing the Interpersonal Gaze Processing model.

**FIGURE 3:**
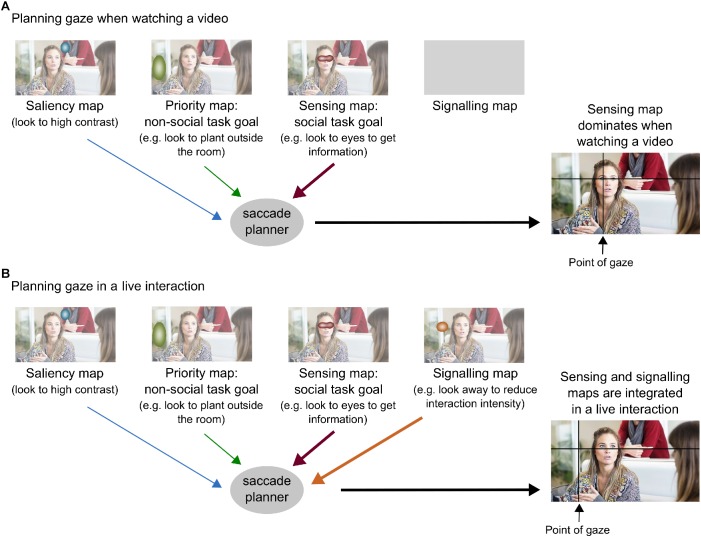
The Interpersonal Gaze Processing model in a social scene. **(A)** Planning gaze when watching a video. **(B)** Planning gaze in a live interaction. Blurbs indicate areas of high saliency depending on the type of map. Original image published by Max Pixel under the Creative Commons CC0 License. Original maps were obtained with Saliency Toolbox for Matlab (Walther and Koch, 2006).

The Interpersonal Gaze Processing model considers how gaze transitions from one state to the other (i.e., how eye movements are planned) when presented with social stimuli (Figure 2,3). This model distinguishes between two situations that differ in the belief in being watched: one where the social stimulus is a picture or video (i.e., cannot see us), and one where the social stimulus is a real person in front of us (i.e., can see us).

In the first case, where the stimulus is a picture or video of another person, there is no need to send a signal because it will not be perceived. Thus, the planning of eye movements only responds to active sensing, which aims to gain maximal information from the stimulus (Yang et al., 2016). The Interpersonal Gaze Processing model considers that gaze patterns derived from active sensing correspond to baseline gaze behavior. When the goal is to get social information from the picture or video (e.g., what is the man in the picture feeling?) gaze patterns will be mostly influenced by sensing maps (see Figure 2,3A). This baseline sensing map reveals how people use gaze to gain different types of social information during interactions. For example, in a noisy environment where it is hard to hear, they will look more to the center of the face to help with speech comprehension; conversely, to recognize emotions they will look more to the eyes (Buchan et al., 2007, 2008; Lewkowicz and Hansen-Tift, 2012). This also demonstrates how task goals (e.g., speech comprehension or emotion recognition) translate in different eye movements depending on the information that needs to be maximized.

**FIGURE 4:**
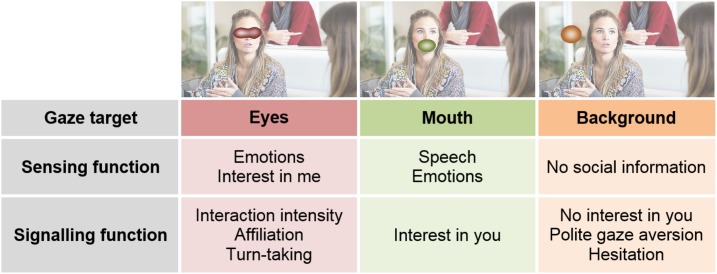
Different sensing and signaling maps may be used in different contexts. Original image published by Max Pixel under the Creative Commons CC0 License. Original maps were obtained with Saliency Toolbox for Matlab (Walther and Koch, 2006).

In the second case, where the stimulus is a real person in front of us, our eyes will be sending a signal to the other person. Here, the Interpersonal Gaze Processing model proposes that gaze patterns result from a trade-off between sensing maps and signaling maps (see Figure 2,3B). This means that the planning of eye movements combines the maximal gain of information from a particular location in the sensing map (e.g., eyes of the other person), together with the optimal benefit of gazing to that location in the signaling map. Figure 4 illustrates how different possible gaze targets on the face of the man can provide various types of information to the woman (sensing function), but also can send different signals to the man (signaling function). Comparing baseline gaze behavior in a video to gaze behavior in a matched real-life interaction, can provide a measure of the signaling components of eye gaze. For example, some studies show that people direct gaze to the eyes of a stranger in a video, but not to the eyes of a live stranger: this indicates that averting gaze from the real person has a meaningful signaling value, since it expresses no desire to affiliate with the stranger and reduces the intensity of the interaction (Argyle and Dean, 1965; Foulsham et al., 2011; Laidlaw et al., 2011). This example considers the case of watching a stranger with a rather neutral face, but another interesting situation is that where partners show emotional facial expressions. Although this scenario has not yet been tested, it would give further insight on how sensing maps and signaling maps are integrated during gaze planning. Moreover, we acknowledge there may also be changes in arousal in association with being watched by a live person (Zajonc, 1965; Myllyneva and Hietanen, 2015; Lyyra et al., 2018), but these effects are not included in our model because of non-specific predictions on sensing and signaling maps.

Thus, the Interpersonal Gaze Processing model proposes that, moment-by-moment, the gaze control systems in the brain must evaluate both the information gained and the signaling potential of a saccade, to determine where to look next. This model and other theories of the audience effect (i.e., Watching Eyes model and reputation management theory) are linked because they are all modulated by the belief in being watched. The Watching Eyes model and reputation management theory explain how the presence of an *observer* modulates an individual’s self- and other-focused cognitive processing, but they do not attempt to explain the dynamics of eye gaze in live *communicative exchanges*. By contrast, the Interpersonal Gaze Processing model places special emphasis on communicative purpose and coordination with other social signals (e.g., other’s gaze direction, speech, facial expressions): while communicative purpose (together with the belief in being watched) is key to define the signaling map, the coordination with other social signals modulates this map on a moment-by-moment basis. Future studies on gaze processing should try to elucidate how each of these factors modulates gaze sensing and signaling during communication, as well as if and how these maps are computed and integrated in the brain.

## Gaze Processing in Autism

Autism Spectrum Condition (ASC) is a developmental condition characterized by difficulties in interpersonal interaction and communication, as well as the presence of restricted and repetitive patterns of behavior (American Psychological Association, 2013). Since eye gaze has a critical role in regulating social interactions and enabling successful communicative exchanges, it is not surprising that the presence of abnormal gaze patterns is one of the most used diagnostic criteria for ASC from early infancy (Zwaigenbaum et al., 2005). Although research into gaze behavior in autistic adults has identified some general patterns, it has also yielded some inconsistent findings: some studies using pictures and videos suggest that they avoid looking at the eyes, whereas others indicate that they have typical gaze patterns (Falck-Ytter and Von Hofsten, 2011; Chita-Tegmark, 2016; Frazier et al., 2017). Some of these discrepancies may be a consequence of the wide spectrum in autistic individuals, but in line with the second-person neuroscience framework (Schilbach et al., 2013; Schilbach, 2016), it has been suggested that they could also be a consequence of the lack of experimental paradigms for studying eye gaze in real social interactions (Chevallier et al., 2015; Von dem Hagen and Bright, 2017; Drysdale et al., 2018). Moreover, a recent qualitative study highlights that self-declared autistic adolescents and adults struggle with the appropriate use and timing of eye gaze during face-to-face interactions (Trevisan et al., 2017). These findings suggest that to fully understand autistic social cognition it is necessary to examine how they process social signals in real dynamic interactions.

### Audience Effects in Autism

We have previously presented two distinct cognitive theories to explain audience effects: the Watching Eyes model (Conty et al., 2016) and reputation management theory (Emler, 1990; Resnick et al., 2006; Tennie et al., 2010). Both theories involve mentalizing and distinction between self-beliefs and other-beliefs, either to process the perceptual state of the observer (Teufel et al., 2010b) or to further infer what the observer thinks of us (Izuma et al., 2010; Cage, 2015). This means that mentalizing is a key cognitive component of audience effects (Hamilton and Lind, 2016). Difficulties in processing mental states of others is one of the hallmarks of autism: they have trouble inferring beliefs and intentions of other people (Happé,, 1994; White et al., 2009), as well as attributing a social meaning to eye gaze (Baron-Cohen et al., 1997), especially when they need to do so spontaneously (Senju et al., 2009). Thus, impaired mentalizing in autistic people implies that being watched will elicit less self-related processing and reputation management, and they will show reduced audience effects (Hamilton and Lind, 2016).

To our knowledge, no studies have directly tested the Watching Eyes model on autistic individuals, but instead have looked at differences in self-referential processing between typical and autistic populations. Lombardo et al. (2007) used a task measuring self-referential memory and found that high-functioning autistic individuals as well as with Asperger Syndrome (two similar subgroups within the autism spectrum) had smaller self-referential bias compared to typical individuals. Moreover, from early infancy autistic individuals’ show reduced orienting to their name, which is a salient stimulus uniquely related to oneself (Werner et al., 2000; Nadig et al., 2010). These studies suggest that autistic people have a general impairment in processing self-related information as distinct from other-related information, already when they are in a non-interactive environment. Interestingly, it has recently been suggested that autistic people might have a narrower cone of direct gaze (i.e., the range of gaze directions that an individual judges as being directed to oneself), which means that they might be less likely to perceive that an observer is watching them (Gianotti et al., 2018). Thus, a plausible prediction is that autistic individuals will fail to process self-relevant signals in interactive environments, such as the belief in being watched (Conty et al., 2016). Studies directly testing effects of being watched on self-referential processing will be needed to clarify this question.

In contrast, a body of research has investigated reputation management in autism. Using the donation task, it has been found that the frequency of donations of autistic participants is not affected by the presence or absence of a confederate who is watching them (Izuma et al., 2011; Cage et al., 2013). It is worth noting that Izuma et al. (2011) found a social facilitation effect in autistic participants on a perceptual task, which indicates that autistic people have specific difficulties with reputation management processes. Cage et al. (2013) further showed that, while typical participants donated more frequently when the observer could reciprocate, autistic participants had reduced expectation of reciprocity. Moreover, autistic children do not engage in flattery behavior toward others (Chevallier et al., 2012) and do not use strategic self-promotion when describing themselves in front of an audience (Scheeren et al., 2010). These findings demonstrate that autistic people are less inclined to manipulate beliefs of observers to maintain their reputation, either due to mentalizing impairments (Frith, 2012) or to social motivation deficits (Chevallier et al., 2013).

However, it is not clear how social norms of eye gaze (i.e., civil inattention; (Goffman, 1963) are implemented in autism, since no study has directly contrasted gaze patterns of autistic individuals in live versus pre-recorded non-communicative interactions. A study by von dem Hagen and colleagues approached this question in typical individuals with high and low autistic traits (Von dem Hagen and Bright, 2017, Experiment 1). Participants were shown videos of a confederate and were deceived to believe that the videos were either pre-recorded or a live video-feed. They found that people with low autistic traits decreased the amount of gaze directed to the face of the confederate in the live video-feed condition, but no reduction was found in the group with higher autism traits. This finding indicates some degree of insensitivity to the belief in being watched and, consequently, to social norms associated with social behavior toward strangers. However, it remains to be seen whether these findings are true for individuals with an ASC diagnosis.

### Interpersonal Dynamics of Gaze in Autism

Few studies have looked at how gaze patterns differ between typical and autistic groups during interactions with communicative purpose, and the evidence is mixed. For instance, when asked to actively engage in an interaction (QandA task) over a video-feed, individuals with high autistic traits looked less toward the face of the confederate than individuals with low autistic traits (Von dem Hagen and Bright, 2017, Experiment 2). Using a similar QandA task in a face-to-face interaction, it was found that high amount of autistic traits was not associated to reduced looking to the face, but to reduced visual exploration (Vabalas and Freeth, 2016). However, in a study testing a sample with autism diagnosis, no differences in visual exploration were found between typical and autistic groups (Freeth and Bugembe, 2018). It is worth noting that in all these studies they found no between-group differences in gaze patterns during speaking and listening periods (i.e., typical/low autistic traits and high autistic traits behave equally), which suggests that to some extent social functions of gaze are preserved in autism (e.g., regulating turn-taking during conversation).

We previously argued that in communicative encounters (direct) eye gaze needs to coordinate with other verbal and non-verbal signals, within and between agents, to successfully exchange information (Bavelas et al., 2002; Trujillo et al., 2018). Several studies indicate that autistic individuals do not use direct gaze as a signal to coordinate intra- and inter-personal social behavior in the same way that typical participants do. Using non-interactive stimuli, it has been shown that autistic adults do not follow gaze after eye contact as much as typical participants (Böckler et al., 2014). Moreover, while in typical individuals direct gaze reduces reaction times to generate an action (Schilbach et al., 2012) or to mimic an action (Forbes et al., 2017), this effect is not found in ASC. Similarly, when participants interact with a virtual avatar that displays contingent gaze patterns, autistic children show less gaze following (Little et al., 2017) and individuals with high autistic traits engage in less facial mimicry following joint attention than individuals with low autistic traits (Neufeld et al., 2016). These findings suggest that reduced coordination between eye gaze and other social behavior may have an impact on the successful progression of the interaction.

A reason why autistic people show poor coordination of social behavior could stem from difficulties in appropriately adjusting gaze to the dynamics of communication. It has been found that infants at high risk for ASC alternate less between initiating and responding to joint attention compared to infants at low risk (Thorup et al., 2018), and that they preferentially orient toward a person that always responds in the same way over a person that can show variable responses (Vernetti et al., 2017). This means that, since early infancy, individuals at high risk for ASC experience less dynamic social contexts and less variety in gaze-contingent events. Using a gaze-contingent eye-tracking paradigm with virtual avatars, Caruana et al. (2017) have found that autistic adults are less accurate and take a longer time than typical adults to respond to joint attention. In line with this, Freeth and Bugembe (2018) have found that when a confederate directly gazes at participants during a QandA task, autistic adults look less at the confederate’s face than typical adults. These findings suggest that difficulties in adjusting eye contact make it hard for autistic individuals to keep pace with rapid and spontaneous face-to-face interactions.

It has been suggested that a lack of exposure to contingent eye gaze in infancy can impact the specialization of brain areas related to gaze processing (Vernetti et al., 2018). Indeed, a study using live video-feed found that some regions in the social neural network (superior temporal sulcus and dorsomedial prefrontal cortex) are equally engaged during periods of joint attention and periods of no joint attention in ASC (Redcay et al., 2012). This is corroborated by previous studies using non-interactive stimuli, where they found abnormal activation of the social neural network (e.g., superior temporal sulcus, right temporo-parietal junction) when autistic adults processed social information conveyed by eye gaze (Pelphrey et al., 2005; Zilbovicius et al., 2006; Philip et al., 2012; Georgescu et al., 2013). Moreover, a hyperscanning study using live video-feed (Tanabe et al., 2012) found that inter-brain coherence (in frontal regions) during eye contact was lower in autistic-typical dyads compared to typical-typical dyads, which might reflect difficulties in processing and integrating social signals in ASC. Thus, these studies suggest that atypical intra- and inter-individual patterns of neural activity in response to direct gaze may underlie difficulties in detecting, processing and sending social signals in autism.

Overall, these findings indicate that autistic individuals have difficulties with social dynamics of gaze in real interactions. However, current research is not enough to clearly distinguish which cognitive components of eye gaze processing are most disrupted in autism. In this sense, the Interpersonal Gaze Processing model (Figure 2,3) provides common ground where studies manipulating various gaze-related factors can come together. We previously suggested that comparing gaze patterns in a video versus a matched real-life interaction provides a measure of the signaling components of eye gaze. If autistic people do not engage in social signaling, the Interpersonal Gaze Processing model predicts that their gaze patterns in live and video conditions should be similar, which is in line with recent evidence (Von dem Hagen and Bright, 2017). Future research should try to systematically study which factors modulating gaze signaling make interpersonal gaze processing challenging in autism.

## Conclusion

Natural social interactions are characterized by complex exchanges of social signals, so achieving successful communication can be challenging. This paper aimed to review research manipulating three key factors that modulate eye gaze processing during social interactions: the presence of an interacting partner who can perceive me, the existence of communicative purpose, and the development of interpersonal and temporal dynamics.

Current findings indicate that the belief in being watched has a strong impact on other-focused social cognition (both on prosocial behavior and social norms of eye gaze), but evidence is less clear for self-focused cognition: future studies should clarify to what extent being watched affects different forms of self-related processes. We also find that, to achieve successful communication, eye gaze needs to coordinate with verbal and non-verbal social signals, both within and between interacting partners. We propose the Interpersonal Gaze Processing model as a framework where gaze sensing and signaling are combined to determine where the eyes will look next in a live interaction. In this model, the belief in being watched and the communicative purpose of the interaction are key to define the gaze signaling map, while the contingencies between different signaling modalities (e.g., gaze, speech) are critical in changing this map on a moment-by-moment basis. Systematic manipulation of these factors could help elucidate how they relate to each other to enable successful communicative encounters, as well as how signaling maps are computed in the brain.

Finally, research on autistic individuals reveals that they are less sensitive to the belief in being watched, but more studies are needed to clarify how the presence of an audience impacts self-related processing in autism. Although evidence on interpersonal dynamics is mixed, it is agreed that autistic individuals have difficulties with social dynamics of eye gaze during real interactions. We argue that the Interpersonal Gaze Processing model provides a framework for future studies to systematically characterize which aspects of gaze communication are most challenging for autistic people.

## Author Contributions

RC wrote the initial draft of the manuscript and prepared the figures. AH made critical revisions to the original draft. RC and AH approved the final version of the manuscript.

## Conflict of Interest Statement

The authors declare that the research was conducted in the absence of any commercial or financial relationships that could be construed as a potential conflict of interest.
